# Focusing on fast food restaurants alone underestimates the relationship between neighborhood deprivation and exposure to fast food in a large rural area

**DOI:** 10.1186/1475-2891-10-10

**Published:** 2011-01-25

**Authors:** Joseph R Sharkey, Cassandra M Johnson, Wesley R Dean, Scott A Horel

**Affiliations:** 1 Program for Research in Nutrition and Health Disparities, School of Rural Public Health, Texas A&M Health Science Center, College Station, TX USA; 2 Program on GIS and Spatial Statistics, School of Rural Public Health, Texas A&M Health Science Center, College Station, TX USA

## Abstract

**Background:**

Individuals and families are relying more on food prepared outside the home as a source for at-home and away-from-home consumption. Restricting the estimation of fast-food access to fast-food restaurants alone may underestimate potential spatial access to fast food.

**Methods:**

The study used data from the 2006 Brazos Valley Food Environment Project (BVFEP) and the 2000 U.S. Census Summary File 3 for six rural counties in the Texas Brazos Valley region. BVFEP ground-truthed data included identification and geocoding of all fast-food restaurants, convenience stores, supermarkets, and grocery stores in study area and on-site assessment of the availability and variety of fast-food lunch/dinner entrées and side dishes. Network distance was calculated from the population-weighted centroid of each census block group to all retail locations that marketed fast food (*n *= 205 fast-food opportunities).

**Results:**

Spatial access to fast-food opportunities (FFO) was significantly better than to traditional fast-food restaurants (FFR). The median distance to the nearest FFO was 2.7 miles, compared with 4.5 miles to the nearest FFR. Residents of high deprivation neighborhoods had better spatial access to a variety of healthier fast-food entrée and side dish options than residents of low deprivation neighborhoods.

**Conclusions:**

Our analyses revealed that identifying fast-food restaurants as the sole source of fast-food entrées and side dishes underestimated neighborhood exposure to fast food, in terms of both neighborhood proximity and coverage. Potential interventions must consider all retail opportunities for fast food, and not just traditional FFR.

## Background

For a variety of economic and social reasons such as cost, convenience, and time-saving, individuals and families are relying more on food prepared outside the home as a source for at-home and away-from-home consumption than in the past [1-5]. Foods from fast-food restaurants dominate away-from-home food consumption, tend to be energy-dense or high in calories and fat, and are associated with poorer diet [6-9]. Research evidence implicates consumption of energy-dense fast food in the obesity epidemic [6,10-12]. However, simply focusing on individual-level consumption of fast food is not enough when considering the documented influence of the food environment on diet and health outcomes [13-17].

Many studies show that the food environment landscape in socioeconomically and geographically disadvantaged areas tends to have easier access, both in terms of proximity (distance to the nearest location) and coverage (number of different locations within a specific area) to fast-food restaurants and small food stores (e.g., convenience and corner stores) than to supermarkets, which offer a selection of healthy foods at lower prices [18-33]. However, restricting the estimation of fast-food access to fast-food restaurants may underestimate potential spatial access to fast-food entrées and side dishes [34]. Notably, in examining the availability of healthier fast-food options, Creel and colleagues (2008) appear to provide the only study that provides a complete picture of the fast-food environment by examining multiple retail sources for fast-food items; namely, traditional fast-food restaurants, convenience stores, and supermarkets/grocery stores. They found that convenience stores and supermarkets/grocery stores provided more than double the potential availability of fast foods in a large rural area than provided by traditional fast-food restaurants alone [34].

The findings of Creel and colleagues suggest that assessments relying exclusively on traditional fast-food restaurants provide an incomplete picture of the fast-food environment and underestimate the exposure to fast food, especially in rural areas where there is relatively greater availability of convenience stores [25,26,34,35]. Recent review articles also have emphasized the need to focus on fast food and assess fast-food exposure from "all outlets" in order to more accurately describe associations between access to fast food and outcomes [12,36]. Others have recognized that not capturing the complete picture of fast-food availability limits associations with fast-food access [20]. In rural areas, where there are limited options for obtaining healthy food, it is important to understand the true exposure to fast food [24,26,29,30,37,38]. Although prior studies estimated spatial access from socioeconomically deprived neighborhoods to retail food stores and the availability of healthier fast-food options from fast-food restaurants and retail food stores [25,34], this study expands our understanding of potential access to fast food and is apparently the first study to: 1) determine potential spatial access to fast-food entrées and side dishes from traditional fast-food restaurants, convenience stores, and supermarkets/grocery stores, using measures of proximity (network distance to the nearest location) and coverage (number of different locations within a specific network distance) in a large rural region of Texas; 2) ascertain the potential spatial access to healthier fast-food options; and 3) examine the relationship between neighborhood deprivation and spatial access to all fast-food opportunities and to fast-food opportunities that offer healthier options.

## Methods

### Geographic setting

The study used data from the 2006 Brazos Valley Food Environment Project (BVFEP), which was approved by the Institutional Review Board at Texas A&M University, and the decennial 2000 U.S. Census Summary File 3 (SF-3) for six rural counties in the Central Texas Brazos Valley region. These counties, which are considered rural based on population density, consist of 101 Census block groups (CBGs) with five urban clusters (population >2,500), cover a land area of 4,466 mi^2^, and include a population of 119,654 people [39-41].

### Data Collection

The BVFEP used ground-truthed methods in a two-stage approach to determine residents' access to fast food and availability of healthier fast-food options in the 101 rural CBG [25,34]. In the first stage, trained observers systematically drove all highways (Interstate, U.S., and State), farm-to-market roads, and city or town streets/roads within the study area. All traditional (supercenters, supermarkets, and grocery stores), convenience (convenience stores and food marts), and non-traditional (dollar stores, mass merchandisers, and pharmacies) food stores, full-service restaurants, and fast-food restaurants were enumerated through direct observation and on-site determination of geographic coordinates using a Bluetooth Wide Area Augmentation System (WAAS)-enabled portable Global Positioning System (GPS) receiver and the World Geodetic System 1984 datum [25,35]. In the second stage, an observational survey instrument was developed, tested, and administered in all fast-food restaurants and food stores by trained observers to determine the availability and variety of fast-food entrées and side dishes and healthier options for fast-food entrées and side dishes [34].

Criteria for identifying healthier options for 11 lunch/dinner entrées and side dishes based on menus, menu boards, or in-store signs have been described in detail [34]. Briefly, 261 fast-food restaurants, traditional food stores (supercenters, supermarkets, and grocery stores), and convenience stores were surveyed; 205 fast-food opportunities were identified, and included 84 (41%) fast-food restaurants, 12 (5.8%) traditional food stores, and 109 (53.2%) convenience stores [34]. Healthier lunch/dinner options included grilled meat, chicken, fish, or other cooked meats; 100% whole wheat or whole grain bun, pizza crust, tortilla, or wrap; lean cuts of other cooked meats, cold cuts, or meat salads; low-fat cheese; and low-fat or fat-free dressing or sauce. When at least one healthier option was identified, a lunch or dinner entrée was classified as having a healthier option [34]. The number of different lunch or dinner entrées with at least one healthier option was summed to create a variety score. Based on the distribution of healthier variety scores, a two-category healthier variety of lunch/dinner entrées was constructed: low variety (first and second tertiles, scores 0/1 entrée) and high variety (highest tertile, scores ≥2 different entrées). The availability of healthier side dish options included: fruit (either without added fat or sugar, or 100% fruit juice); vegetables that were either steamed/roasted or not fried; potatoes that were baked, no fat added, or available with low-fat options; soup identified as low-fat or reduced sodium; baked chips; salads with low-fat dressing; corn without fat or without sauce; or chili with lean meat or turkey [34]. Using the approach described for lunch or dinner entrées, a variable for the variety of lunch or dinner side dishes with healthier options was dichotomized as ≥2 different lunch/dinner side dishes vs. 0/1 lunch/dinner side dishes.

### Neighborhood socioeconomic deprivation

Neighborhood socioeconomic deprivation was based on seven characteristics from the SF-3 at the level of the CBG: neighborhood unemployment, poverty-level income, low education attainment (persons ≥25 y with less than a 10^th^-grade education), household crowding (occupied households with >1 person per room), households receiving public assistance, households with no available vehicle, and occupied housing with no telephone service. A three-category neighborhood socioeconomic deprivation variable was constructed: low deprivation (highest overall in socioeoconomics and lowest quartile of weighted and standardized deprivation scores), medium deprivation (middle two quartiles), and high deprivation (lowest overall in socioeconomics and highest quartile of deprivation scores) [25,38].

### Spatial access to fast food

The road network distance was calculated with ESRI's Network Analysis extension in ArchInfo 9.2, which computed the distance along the road network between paired point data (population-weighted centroid of each CBG and the geographic position in front of each fast-food opportunity within the study area) [25]. The population-weighted centroid was calculated using the ArcGIS Desktop Tool Mean center (Version 9.2, Environmental Systems Research Institute) [38]. Two criteria of potential spatial access were calculated from each CBG: 1) proximity and 2) coverage [38,42]. Proximity is used to measure distance to the nearest fast-food restaurant or food store. Coverage adds the dimension of variety and competition within a specified distance and is not limited to the fast-food restaurants or food stores within an administratively-defined area, such as CBG.

#### Proximity

Separate network distances in miles were calculated from the population-weighted centroid of each CBG to the nearest fast-food restaurant, fast-food opportunity, fast-food opportunity with a variety of healthier lunch/dinner entrées, and fast-food opportunity with a variety of healthier lunch/dinner side dishes within the six-county study area[38]. Network data were provided by the 2003 Tele Atlas Dynamap Transportation version 5.2.

#### Coverage

Network Analyst computed the total number of fast-food restaurants, fast-food opportunities, fast-food opportunities with a variety of healthier lunch/dinner entrées, and fast-food opportunities with a variety of healthier side dishes within one, three, and five miles, using the shortest network distance from the population-weighted centroid of each CBG. Proximity measured the shortest distance to the nearest location, while coverage indicates the number of purchase opportunities [38]. In combination, a shorter distance and greater number of opportunities suggest greater potential spatial access to fast food.

### Statistical Analysis

All statistical analyses were conducted with Release 11 of Stata Statistical Software (College Station, TX, 2009); *p *< 0.05 was considered statistically significant. Descriptive statistics were calculated for the availability of a variety of healthier lunch/dinner entrée and side dish options by type of fast-food opportunity (fast-food restaurant, traditional food store, and convenience store). Comparisons between store types were examined using two-way tables of frequency, along with the Fisher's exact test. Proximity and coverage were calculated for the overall 101 CBG and by level of neighborhood socioeconomic deprivation. Nonparametric tests for trend were estimated across categories of increasing neighborhood deprivation [43]. Distance to the nearest fast-food restaurant was compared with the distance to the nearest fast-food opportunity, distance to nearest fast-food opportunity with a variety of healthier lunch/dinner entrée options, or nearest fast-food opportunity with a variety of healthier lunch/dinner side dish options by testing for equalities in mean, median, and distribution of distance measures, using Wilcoxon Matched-Pairs signed-rank test. The null hypothesis was that there is no difference between the distributions. Coverage differences were also examined using the Wilcoxon Matched-Pairs signed-rank test. Finally, four multivariate regression models were fitted to determine the relationship between neighborhood deprivation and potential spatial access to fast food, controlling for population density: 1) proximity, 2) one-mile coverage, 3) three-mile coverage, and 4) five-mile coverage. In multivariate regression, several dependent variables were jointly regressed on the same independent variables. The multivariate model approach was chosen instead of four separate multiple regression models (one for each outcome variable) for two reasons: 1) the four outcome variables were correlated with each other and the multivariate regression accounted for this correlation when testing hypotheses about the predictor variables; and 2) the final collection of models was easier to interpret if the same predictor variables are identified [38].

## Results

Almost 87% (*n *= 73) of fast-food restaurants, 68% (*n *= 74) of convenience stores, and 84% (*n *= 10) of traditional food stores offered a healthier option in ≥1 lunch/dinner entrée. Healthier options in ≥2 different entrées were observed in 65.5% (*n *= 55) fast-food restaurants, 19.3% (*n *= 21) convenience stores, and 58.3% (*n *= 7) traditional food stores. While fast-food restaurants and traditional food stores were more likely to offer healthier options in ≥2 different options than convenience stores (*p *< 0.001), there was not a statistically significant difference between fast-food restaurants and traditional food stores (*p *= 0.749). Healthier options in ≥1 side dish were observed in almost 35% (*n *= 29) fast-food restaurants, 11% (*n *= 12) convenience stores, and 75% (*n *= 9) traditional food stores. A variety (≥2 side dishes with a healthier option) were found in 8.3% (*n *= 7) fast-food restaurants, 4.6% (*n *= 5) convenience stores, and 41.7% (*n *= 5) traditional food stores. A greater proportion of traditional food stores compared with fast-food restaurants offered healthier options in a variety of side dishes (*p *< 0.001), and there was no statistically significant difference between fast-food restaurants and convenience stores (*p *= 0.371).

### Potential spatial access to fast food

**Figure 1:**
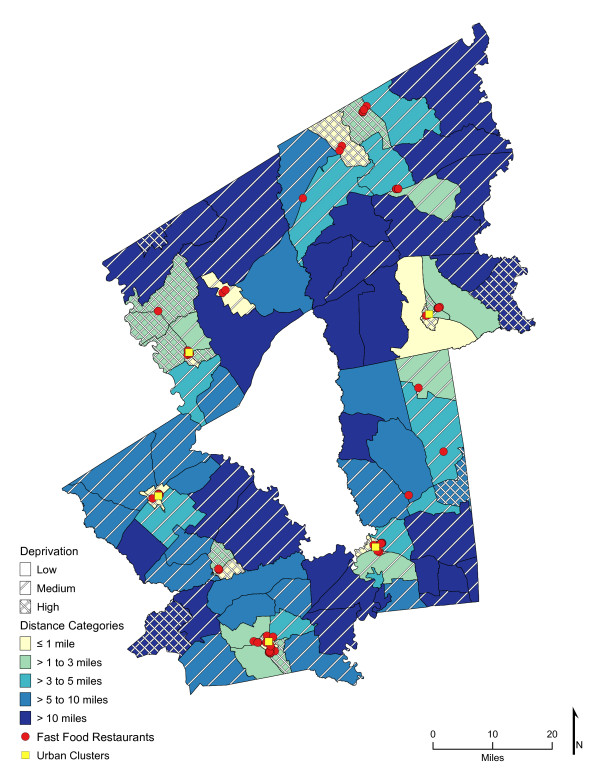
**Area-level (CBG) Deprivation and Spatial Access (Proximity) to Fast-Food Restaurants**.

**Figure 2:**
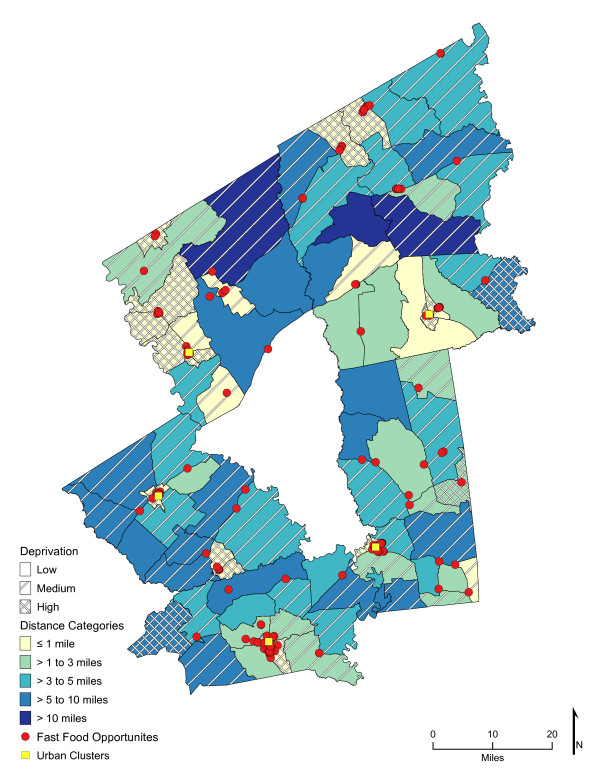
**Area-level Deprivation and Spatial Access (Proximity) to Fast-Food Opportunities**.

**Figure 3:**
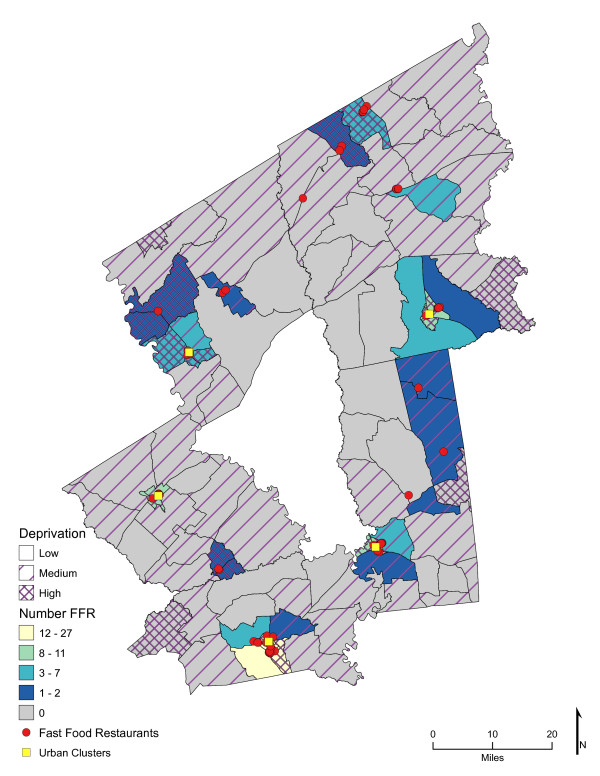
**Area-level Deprivation and Spatial Access (Coverage within 3 Miles) to Fast-Food Restaurants**.

**Figure 4:**
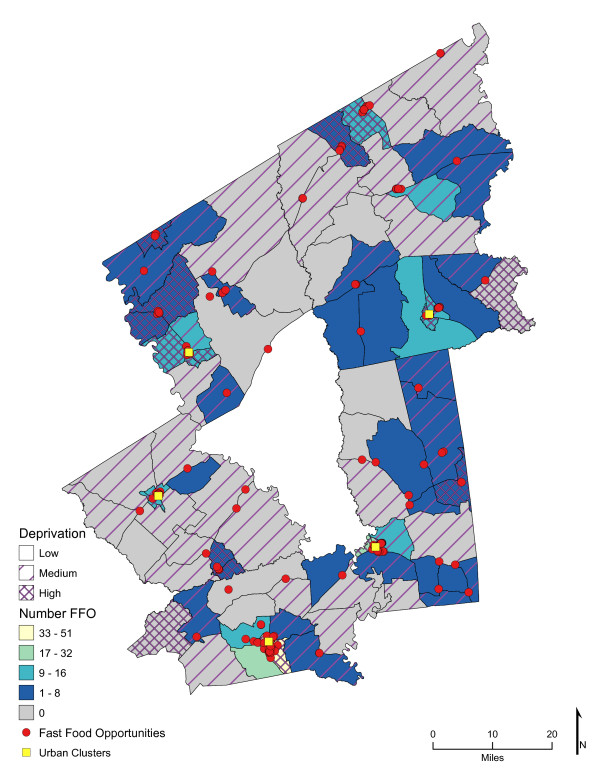
**Area-level Deprivation and Spatial Access (Coverage within 3 Miles) to Fast-Food Opportunities**.

**Figure 5:**
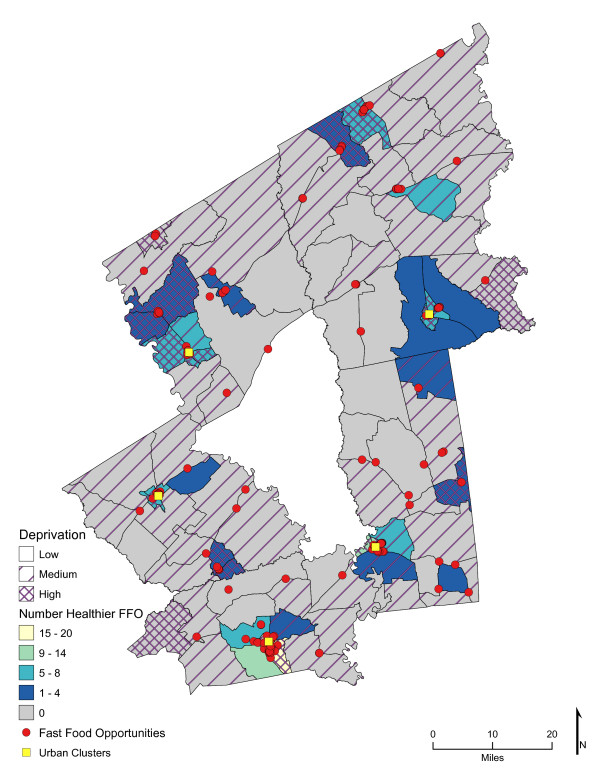
**Area-level Deprivation and Spatial Access (Coverage within 3 Miles) to Healthier Fast-Food Options**.

Chloropleth maps (Figure 1 and figure 2) illustrate the spatial distribution of access to fast-food restaurants and fast-food opportunities and neighborhoods with low, medium, and high socioeconomic deprivation. The lightest color area indicates CBG that are one-mile or closer one-way to the nearest fast-food restaurant (25.7%, *n *= 26; and 59% of high deprivation neighborhoods) or fast-food opportunity (30.7%, *n *= 31; and 74% of high deprivation neighborhoods). Distances calculated from the population-weighted centroid to the nearest fast-food restaurant were greater (*p *< 0.001) than those calculated to the nearest fast-food opportunity (**Table **1). For example, the median distance to the nearest location for fast-food lunch or dinner entrées was 4.5 miles if the location was a fast-food restaurant and 2.7 miles if it represented the nearest fast-food opportunity (fast-food restaurant, convenience store, or traditional food store). In over 73% of the neighborhoods (*n *= 74 CBG), the distance to nearest fast-food restaurant was greater than to the nearest fast-food opportunity (range of <0.1 mile to 17 miles), and all CBG had better access to a fast-food opportunity than to a fast-food restaurant. Similarly, coverage or the exposure to fast food among multiple fast-food opportunities was greater than to fast-food restaurants alone (*p *< 0.001). Chloropleth maps (Figure 3 and Figure 4) illustrate neighborhood coverage for fast-food restaurants and fast-food opportunities within three network miles (darker the color, the greater the number of locations). There were 12 CBG (6 were high deprivation) with at least twelve different fast-food restaurants within three miles and 32 CBG (19 were high deprivation) with at least twelve fast-food opportunities. Figure 5 shows neighborhood socioeconomic deprivation and coverage for healthier fast-food options within three miles; there were 17 CBG (10 were high deprivation) with at least twelve fast-food opportunities with healthier options. Table 1 shows the distribution (mean, standard deviation, and median) of proximity and coverage measures for fast-food restaurants and fast-food opportunities by level of neighborhood socioeconomic deprivation for the overall study area of 101 CBG. Proximity and coverage within one, three and five miles of population-weighted centroids increased across levels of increasing neighborhood deprivation (*p *< 0.001). The greater the neighborhood deprivation, the better the access to the nearest fast-food restaurant, fast-food opportunity, or healthier options in lunch/dinner entrées or side dishes and the greater the number of opportunities to purchase fast food, especially lunch/dinner entrées or side dishes with healthier options. In addition, the increase in access, through both proximity and coverage for fast-food opportunities compared with fast- food restaurants, was observed for each of the three categories of neighborhood deprivation. Specifically, the most socioeconomically deprived neighborhoods had significantly better spatial access to fast-food opportunities that offered healthier options than less deprived areas (*p *< 0.001).

**Table 1:** Spatial accessibility to fast-food restaurants and fast-food opportunities by neighborhood socioeconomic deprivation, using measures of proximity and coverage^1,2^

	All Deprivation (*n *= 101)	Low Deprivation (*n *= 26)	Medium Deprivation (*n *= 48)	High Deprivation (*n *= 27)
**SPATIAL ACCESSIBILITY**				
***Proximity, mi***				
Fast-food restaurant	6.2 ± 5.5 (4.5)	9.1 ± 5.0 (9.5)	7.0 ± 5.3 (6.1)	2.1 ± 3.7 (0.7)^¶^
Fast-food opportunity	3.3 ± 3.0 (2.7)	5.2 ± 3.6 (4.3)	3.5 ± 2.4 (3.7)	1.0 ± 1.6 (0.6)^¶^
Healthier entrée (≥1)	3.7 ± 3.3 (4.3)	5.5 ± 3.6 (4.7)	4.2 ± 3.0 (3.8)	1.1 ± 1.5 (0.6) ^¶^
Healthier entrées	5.7 ± 5.1 (4.3)	7.9 ± 4.9 (7.6)	6.7 ± 4.9 (6.3)	1.8 ± 3.5 (0.7)^¶^
Healthier side dishes	7.3 ± 3.3 (6.5)	10.7 ± 7.8 (4.7)	8.4 ± 4.9 (3.8)	2.3 ± 3.3 (0.6)^¶^
***Coverage - 1 mi***				
Fast-food restaurant	1.6 ± 3.3 (0)	0.7 ± 3.5 (0)	1.2 ± 3.4 (0)	3.0 ± 2.3 (3)^¶^
Fast-food opportunity	3.2 ± 5.7 (0)	1.1 ± 5.5 (0)	2.3 ± 5.4 (0)	7.0 ± 4.7 (7)^¶^
Healthier entrée (≥1)	2.5 ± 4.7 (0)	1.0 ± 4.9 (0)	1.8 ± 4.6 (0)	5.1 ± 3.5 (5)^¶^
Healthier entrées	1.4 ± 2.5 (0)	0.5 ± 2.3 (0)	0.9 ± 2.4 (0)	3.0 ± 2.2 (3)^¶^
Healthier side dishes	0.4 ± 0.9 (0)	0.4 ± 0.2 (0)	0.2 ± 0.8 (0)	1.2 ± 1.0 (1)^¶^
*** Coverage - 3 mi***				
Fast-food restaurant	5.0 ± 8.4 (0)	2.3 ± 6.4 (0)	3.5 ± 7.2 (0)	10.4 ± 9.7 (9)^¶^
Fast-food opportunity	9.9 ± 15.5 (1)	4.6 ± 11.6 (0.5)	7.0 ± 13.5 (1)	20.2 ± 17.8 (14)^¶^
Healthier entrée (≥1)	7.7 ± 12.5 (1)	3.6 ± 9.4 (0)	5.6 ± 10.8 (0.5)	15.6 ± 14.6 (10)^¶^
Healthier entrées	4.2 ± 6.4 (0)	1.8 ± 4.8 (0)	2.9 ± 5.6 (0)	8.7 ± 7.0 (6)^¶^
Healthier side dishes	1.4 ± 2.0 (0)	0.6 ± 1.3 (0)	0.9 ± 1.7 (0)	3.1 ± 2.2 (3)^¶^
*** Coverage - 5 mi***				
Fast-food restaurant	6.1 ± 9.1 (1)	4.0 ± 8.8 (0)	4.8 ± 8.2 (0)	10.6 ± 9.6 (9)^¶^
Fast-food opportunity	12.7 ± 17.2 (3)	8.5 ± 16.6 (1)	10.0 ± 15.4 (2)	21.4 ± 18.3 (15)^¶^
Healthier entrée (≥1)	9.8 ± 13.8 (3)	6.3 ± 13.3 (1)	8.0 ± 12.4 (1.5)	16.4 ± 14.8 (10)^¶^
Healthier entrées	5.3 ± 7.1 (1)	3.3 ± 6.8 (0)	4.2 ± 6.5 (0)	9.3 ± 7.2 (7)^¶^
Healthier side dishes	1.7 ± 2.2 (0)	1.0 ± 1.8 (0)	1.2 ± 2.0 (0)	3.2 ± 2.1 (4)^¶^

^1^Fast-food opportunities include fast-food restaurants, traditional food stores (supercenters, supermarkets, grocery stores), and convenience stores that market fast food lunch/dinner entrées and/or side dishes.

^2 ^Values calculated for each of the 101 CBG (census block group) in the study area. Proximity determined by the network distance from each population-weighted CBG centroid to the nearest fast-food only restaurant or fast-food opportunity; coverage is determined by the number of fast-food restaurants or fast-food opportunities within a specific network-based distance. Distances (proximity), numbers (coverage) are shown as mean ± standard deviation (median) overall and by category of deprivation.

Healthier entrée (≥1) = at least one lunch/dinner entrée with a healthier option; Healthier entrées = at least 2 lunch/dinner entrées with a healthier option; Healthier side dishes = at least 2 side dishes with a healthier option.

Level of statistical significance for test for trend across ordered groups of neighborhood socioeconomic deprivation: ^‡^*p *< 0.05 ^§^*p *< 0.01 ^¶^*p *< 0.001

### Neighborhood deprivation and spatial access to fast food

Independent of population density, which was strongly related to all outcomes (*p *< 0.001), high deprivation neighborhoods had the best potential access in terms of proximity to the nearest fast-food restaurant, fast-food opportunity, variety of healthier entrée options, and variety of healthier side dish options (**Table **2). Table 3 shows the associations between neighborhood deprivation and fast-food coverage, controlling for population density. High neighborhood deprivation was associated with greater coverage access within one-mile to fast-food opportunities, variety of healthier entrée options, and variety of healthier side dish options; and at three miles, high neighborhood deprivation was associated with greater access to multiple fast-food restaurants, fast-food opportunities, opportunities for healthier entrée options, or opportunities for healthier side dish options. At five miles (data not shown), deprivation was only associated with multiple opportunities for variety of healthier side dish options.

**Table 2:** Association between proximity to fast food and area deprivation, using multivariate linear regression model

	Access as network distance to the nearest			
	
	Fast-food restaurant	Fast-food opportunity	Variety healthier entrée options	Variety healthier side dish options
	
Deprivation	b (SE)	b (SE)	b (SE)	b (SE)
High	-5.46 (0.131)^‡^	-3.43 (0.71)^‡^	-4.72 (1.23)^‡^	-6.76 (1.48)^‡^
Medium	-1.89 (1.11)	-1.56 (0.60)^†^	-1.07(1.04)	-2.14 (1.25)

R^2^	0.336	0.345	0.323	0.358
*P*	<0.001	<0.001	<0.001	<0.001

NOTE: The four equations were simultaneously estimated, controlling for population density. Deprivation was entered as categorical variable; low deprivation is referent group. Population density entered as continuous variable. Results reported as multivariate-adjusted b (SE). Statistically significant variables are indicated as: *< 0.05 ^†^< 0.01 ^‡^< 0.001

**Table 3:** Association between 1-mile and 3-mile coverage of fast-food opportunities and area deprivation, using multivariate linear regression model

Model 1	Access (number of fast-food opportunities within 1 network mile)			
	
	Fast-food restaurant	Fast-food opportunities	Variety healthier entrée options	Variety healthier side dish options
	
Deprivation	b (SE)	b (SE)	b (SE)	b (SE)
High	0.24 (0.59)	2.39 (0.90)^†^	1.02 (0.43)*	0.83 (0.18)^‡^
Medium	0.24 (0.50)	0.67 (0.76)	0.23 (0.36)	0.16 (0.15)

R^2^	0.611	0.708	0.662	0.509
*P*	<0.001	<0.001	<0.001	<0.001

	
**Model 2**	Access (number of fast-food opportunities within 3 network miles)			
	
	Fast-food restaurants	Fast-food opportunities	Variety healthier entrée options	Variety healthier side dish options
	
Deprivation	b (SE)	b (SE)	b (SE)	b (SE)

High	2.75 (1.31)*	5.91 (2.42)*	2.92 (0.98)^†^	1.37 (0.35)^‡^
Medium	0.45 (1.11)	1.11 (2.05)	0.49(0.83)	0.11 (0.29)

R^2^	0.713	0.716	0.722	0.658
*P*	<0.001	<0.001	<0.001	<0.001

NOTE: In both models, the four equations were simultaneously estimated, controlling for population density. In model 1 (fast-food opportunities within 1 network mile), deprivation entered as categorical variable; low deprivation is referent group. Model 2 estimates the relationships between deprivation and the number of fast-food opportunities within 3 network miles. In both models, population density entered as continuous variable. Results reported as multivariate-adjusted b (SE). Statistically significant variables are indicated as: *< 0.05 ^†^< 0.01 ^‡^< 0.001

## Discussion

The demand for food prepared outside the home, especially fast food, is increasing. It dominates away-from-home consumption, contributes energy-dense foods to the diet, and has been implicated in the obesity epidemic [2,6-12,44,45]. Although spatial access to fast-food restaurants has been shown to correlate with dietary intake [6,46-48], the picture we have, which focuses primarily on chain fast-food restaurants as the source of fast food, is incomplete and may underestimate the exposure to fast food [36].

This study extends our understanding of potential spatial access to fast food from rural neighborhoods, not just to fast-food restaurants, but to all retail opportunities for fast-food entrées and side dishes [34]. We examined two dimension of spatial access: 1) proximity or the distance to the nearest fast-food restaurant, fast-food opportunity, and fast-food opportunity with a good variety of healthier options, and 2) coverage or the number of fast-food restaurants or opportunities to purchase fast food within a specified distance of the neighborhood [38]. This is apparently the first study, to our knowledge, that evaluates the measurement of the fast-food environment and examines the relationship between neighborhood characteristics and potential spatial access to fast food from all opportunities that market fast food and healthier fast-food options in a large rural area. Our analyses revealed that identifying fast-food restaurants as the sole source of fast-food entrées and side dishes underestimated neighborhood exposure to fast food, and in terms of both neighborhood proximity and coverage. Definitions of the fast-food environment should not be limited to traditional fast-food restaurants, as this can significantly misrepresent neighborhood access to all fast food and to a variety of healthier fast-food options. In addition, residents of high deprivation neighborhoods had relatively better spatial access to all fast food and to a variety of healthier fast-food entrée and side dish options compared to residents of low deprivation neighborhoods.

### Spatial access to fast food

This study builds on the work of Creel and colleagues who were the first to describe nontraditional fast-food opportunities by documenting the presence of fast-food entrées and side dishes in retail food stores (convenience stores, supermarkets, and grocery stores) in a large rural area [34]. In a complete picture of the retail fast-food environment, convenience stores and supermarkets/grocery stores that marketed fast food provided almost 60% of the opportunities to purchase fast food [34]. This has been explained by the recognition by convenience stores of the consumer's preference for convenient shopping, fast service, and longer hours; and for the need of convenience stores to attract and hold customers in the face of increased cost and competition [49-51]. However, little was known regarding the extent to which neighborhood spatial access to fast food differed between traditional fast-food restaurants and fast-food opportunities. We found that spatial access to fast-food entrées and side dishes from fast-food opportunities (traditional fast-food restaurants, convenience stores, or supermarkets/grocery stores) was significantly better than access to traditional fast-food restaurants. The median distance to the nearest fast-food opportunity was 2.7 miles, compared with 4.5 miles to the nearest fast-food restaurant. On the average, there were 3.2 fast-food opportunities within 1 mile and 9.9 within 3 miles of a neighborhood, compared with 1.6 (within 1 mile) and 5.0 (within 3 miles) traditional fast-food restaurants. The addition of product offerings, such as fast foods, to the primary business of convenience stores and supermarkets provides these retail stores with new sales opportunities, and consumers with increased access to fast food[2,34]. This extends prior research, primarily in urban areas, that focused on traditional fast-food restaurants, most often the major fast-food restaurant chains [20,24,36,50,52-57]. Similar relationships in proximity and coverage were observed within each level of neighborhood deprivation. Regardless of definition of fast-food environment (traditional fast-food restaurant or fast-food opportunity), access in terms of proximity and coverage significantly improved across neighborhoods of increasing deprivation. These results overcome the limitations previously identified by researchers when describing fast-food availability [12,20,36]. Our results are consistent with prior work that found better potential spatial access to supermarkets and to food stores with fruit and vegetables for residents of high deprivation rural neighborhoods, compared with low deprivation neighborhoods [25,38], and at odds with a New Zealand study that found more deprived rural areas (using census meshblocks) faced greater distances to multinational fast-food outlets [58]. In fact, the difference in mean and median distances that we observed between high-deprivation neighborhoods and either medium or low-deprivation neighborhoods was extremely large and held for both proximity and coverage, and for fast-food only, fast-food opportunities, and healthier options. As previously observed, one explanation may be that the five urban clusters in our study area (towns with populations of 3,181 - 11,952) had the greatest population density, and clustering of socioeconomic deprivation characteristics, and therefore more fast-food restaurants, convenience stores, and supermarkets/grocery stores [25].

### Spatial availability of healthier fast-food options

Little is known about the availability of healthier options for fast-food entrées and side dishes [34]. Building on the work of Creel and colleagues in the identification of healthier options in rural fast-food opportunities, we found greater spatial access to a healthier option in at least one entrée, compared with access to a traditional fast-food restaurant. This was consistent when considering both proximity and coverage, and within categories of neighborhood socioeconomic deprivation. One explanation may be that some convenience stores and supermarkets/grocery stores provide healthier options for their entrées and/or side dishes [34]. Interestingly, when the number of healthier options increased from one to two, spatial access diminished, and was worse for healthier options in side dishes.

### Neighborhood deprivation, access, and availability

Consistent with prior work indicating a relationship between increasing neighborhood deprivation and better potential spatial access to food stores, this study found significantly better access, in terms of proximity and coverage, to fast-food restaurants, fast-food opportunities, and healthier fast-food options by residents of high deprivation neighborhoods [25]. In unadjusted models, access to the nearest fast-food restaurant and fast-food opportunity improved significantly with increasing neighborhood deprivation. Improved access in terms of the number of different fast-food restaurants or fast-food opportunities within one, three, and five miles of the neighborhood population-weighted center was also observed as neighborhood deprivation increased. Within each category of deprivation, proximity and coverage for both fast-food opportunities was significantly better than to fast-food restaurants. Further, the availability of healthier fast-food options improved with increasing deprivation. Finally, the findings from the multivariate models confirmed that significantly better access to fast food - fast-food restaurants, fast-food opportunities, healthier entrée options, and healthier side-dish options - remained after adjusting for population density.

Key findings from this study add to the discussion and understanding of neighborhood exposure to fast food with or without the availability of healthier options. As previously mentioned, exposure to fast food in the U.S., U.K., Canada, Australia, and New Zealand has been based on fast-food restaurants, primarily national or international chains [12,19-21,26,36,53,55,56,58-62]. At least in rural areas, the opportunities for fast-food entrées and side dishes are greater among nontraditional fast-food outlets, such as convenience stores, supermarkets, and grocery stores. Importantly, in this rural area, reliance on fast-food restaurants as the sole source of fast food would have dramatically underestimated both proximity to the nearest retail source of fast food and coverage in the number of different venues of exposure.

### Strengths

There are several strengths to this study. First, this study relied on identification of all fast-food restaurants and food stores through ground truthing, which has been shown to be more accurate in small-town and rural areas than reliance on secondary or publicly available lists [25,35]. Second, data on presence of fast-food entrées and side dishes and availability of healthier options in fast-food restaurants and all food stores were collected through a comprehensive on-site observational survey, which provides a more complete picture of the fast-food environment. The inclusion of all fast-food opportunities for assessing exposure to fast food overcomes the limitation association with relying on fast-food restaurants alone and provides a more complete picture of the fast-food environment [36].

### Limitations

The data allowed us to determine potential access, but do not provide the necessary information to determine utilization or realized access; that is, where, how frequently, and what fast-food entrées and/or side dishes are purchased by rural residents. As we have previously pointed out, potential spatial access assumes that the fast-food trip originates from the residence, which in this study was the population-weighted centroid of each CBG [25,38]. However, the starting point for fast-food acquisition may vary and depend on time and location of work or other activities in multiple stops [63,64]. Future work with rural families will allow us to understand the influence of traditional and nontraditional sources of fast food in rural areas on overall dietary intake. Another limitation is use of area data from the 2000 U.S. Census, which is now outdated. We plan to update our work with the release of the 2010 U.S. Census at the level of the CBG. Limitations in the data on healthier fast-food options have been noted elsewhere [34]. Finally, we are unable to generalize our findings beyond this rural area. Future research plans call for a similar examination in small-town and rural areas in other regions of Texas and outside Texas.

## Conclusion

Despite these limitations, this study furthers our understanding about access and availability of fast food in a large rural area. This paper responded to methodological challenges that have been identified in measuring potential food access in rural areas [35]. The measurement of the fast-food environment recognized the increased exposure provided by convenience stores, supermarkets, and grocery stores, which have expanded their product categories in response to both business and consumer demands. As we have shown, restricting fast-food exposure to the traditional fast-food restaurants would significantly understate access to fast food and availability of healthier fast-food options. Our use of proximity and coverage provided two dimensions of access; that is, network distance to the nearest location and the number of different locations within one, three, and five miles of the neighborhood. The lack of available healthier options should be considered as an intervention point for improving nutritional health in rural populations, regardless of level of neighborhood deprivation. Considering that food choice and consumption may be constrained by available options, the fast-food environment is central to understanding an individual's acquisition of away-from-home foods and dietary intake[58,65,66]. Potential interventions must consider all retail opportunities for fast food, and not just traditional fast food restaurants.

## Competing interests

The authors declare that they have no competing interests.

## Authors' contributions

JRS developed the original idea for assessing spatial access to fast food. JRS worked on the development of the instrument and the protocol for collection of data. JRS and CMJ wrote the first draft of the paper. SAH was responsible for geocoding and spatial calculations. JRS, CMJ, WRD, and SAH read and approved the final manuscript.
